# Therapeutic Possibilities of Ceftazidime Nanoparticles in Devastating Pseudomonas Ophthalmic Infections; Keratitis and Endophthalmitis

**Published:** 2012

**Authors:** Mehrdad Mohammadpour, Mahmood Jabbarvand, Nasser Karimi

**Affiliations:** 1 Ophthalmology Department and Eye Research Center, Farabi Hospital, Tehran University of Medical Sciences, Tehran, Iran

**Keywords:** Pseudomonas aeruginosa, Keratitis, Endophthalmitis, Nanoparticles

## Abstract

As the number of contact‐lens wearers rises worldwide, Pseudomonas aeruginosa (PA) keratitis is attracting more attention as a major public health issue. Corneal lesions of PA, being the most intimidating complication of contact‐lens wearer, can progress rapidly in spite of local antibiotic treatment, and may result in perforation and the permanent loss of vision. One of the explanations proposed for the evasion of the pathogen from immune responses of the host as well as antibacterial treatment is the fact that invasive clinical isolates of PA have the unusual ability to invade and replicate within surface corneal epithelial cells. In this manner, PA is left with an intracellular sanctuary. Endophthalmitis, albeit rare, is another ophthalmic infection faced by the challenge of drug delivery that can be potentially catastrophic. The present hypothesis is that nanoparticles can carry anti‐pseudomonas antibiotics (e.g. ceftazidime) through the membranes, into the “hidden zone” of the pathogen, hence being an effective and potent therapeutic approach against pseudomonas keratitis and endophthalmitis.

## INTRODUCTION

It has been projected that nearly 125 million people worldwide, wear contact lenses [1]. Microbial keratitis is the most intimidating complication of the contact-lens wearer. The prodigious population at risk brings about this complication as an important public issue. Pseudomonas aeruginosa [PA], a virulent gram-negative bacterium, has persistently been reported as the most frequent aetiology of contact lens-related microbial keratitis. Among people with contact lens keratitis, PA accounts for the largest mean diameter of corneal ulcers, the highest mean number of days in hospital, the greatest mean number of outpatient visits, and the poorest visual acuity outcome [2]. PA is sensitive to ceftazidime, ciprofloxacin, and amikacin [3]. This topical treatment involves applying antibiotic eye drops every 5 min for 1 h and then every 15 to 30 min for 48 h or longer, which is an obviously demanding regimen of treatment that necessitates hospitalisation [4]. Despite topical antibiotic treatment, PA corneal ulcers may progress, and eventually, perforation may occur, making surgical intervention necessary [5]. A new therapeutic approach requiring less frequent applications of topical antibiotics whilst enhancing the efficacy has been the objective of many researches.

The cornea is not the only territory in ophthalmology where PA can be a real challenge. Deeper in the eye the problem gets even more serious. Endophthalmitis is a rare but catastrophic complication of intraocular surgery and penetrating injuries. The frequency distribution of the microorganisms isolated in different case series varies significantly. But in most, if not all, series, a significant relationship between gram-negative isolates [led by PA] and poor visual outcome has been reported [6]. Intravitreal administration of antibiotics, although quite invasive, is the mainstay of treatment of acute endophthalmitis, due to the poor ocular penetration of antibiotics administered by alternative routes. 

Systemically administered drugs have poor access to the retina and vitreous cavity because of the blood-aqueous and blood-retinal barriers. On the other hand, the topical instillation of conventional drugs usually yields low bioavailability. Physiological mechanisms, such as blinking and tear drainage, reduce the residence time of topically-applied drugs. Eye drops placed onto the ocular surface are washed away in less than 30 s after instillation [7, 8]. Furthermore, sealing the corneal epithelium by means of annular tight junctions [zonula occludens] leaves the cornea as a major ocular barrier that is almost impermeable to any substance larger than 500Da [9, 10]. Most of the commonly used topical drugs are larger than that and, consequently, do not cross the cornea. Although most of the commonly used topical drugs can permeate throughout the conjunctiva and the underlying sclera it is generally believed to be a “non-productive passage” [11]. Altogether, the existence of impermeable cell barriers along with the physiological processes permits less than 5% of topically administered drugs to reach intraocular tissues [12].

## HYPOTHESES

**A new solution**

It has been well-established that invasive clinical isolates of PA have the unusual ability to invade and replicate within surface corneal epithelial cells [13]. Since commonly used topical anti-microbial agents [such as aminoglycosides] have a limited penetration into the cornea, PA is left with an intracellular sanctuary [14]. Drug delivery becomes even more difficult in the case of endophthalmitis. Considering the vast variety of novel drug delivery systems which are in our armamentarium nowadays, the following hypothesis is presented: anti-pseudomonas nanoparticles can effectively reduce sequels of pseudomonas keratitis/endophthalmitis. The transformation of conventional dosage forms of antibiotics to nanoparticles and nanosuspensions may improve drug efficacy, safety and bioavailability, as well as patient compliance [15].

## DISCUSSION

Nanoparticles [more properly designated as colloidal carriers] consist of 1µm or smaller particles, suspended in an aqueous solution. This term can refer to micelles, emulsions, nanospheres, nanocapsules and liposomes [16]. Intensive research in recent decades has investigated the effect of colloidal carriers on the transcorneal penetration of a wide variety of therapeutic large, poorly water-soluble molecules. Kassem et al. [17] demonstrated the enhanced absorption and potency of glucocorticoid-associated nanosuspensions instilled into the lower cul-de-sac of rabbits by measuring the increase in intraocular pressure [IOP]. Calvo [18] has shown that colloidal particles of indomethacin were taken up preferentially by the corneal epithelial cells through endocytosis. Resembling a reservoir, the cornea then releases the drug to the adjacent tissues. Calvo and co-workers [19, 20] have also made PCL nanocapsules containing cyclosporine. After topical administration, these capsules were taken up by corneal epithelial cells and achieved corneal levels of cyclosporine that were five times higher than when using a 10mg/ml cyclosporine oily solution. Losa et al. revealed that nanoparticle formulations of amikacin, using Dextran 70000 as a stabiliser, can lead to a significant increase of the antibiotic concentration, not only in the cornea but also in the aqueous humor [21]. Nanoparticle-mediated delivery not only overcomes the corneal epithelium barrier but it can also prolong the residence time of a drug in the pre-corneal tear film layer, hence reducing the dosage frequency [22]. These evidences, among others, support the idea that nanoparticle formulations of antibiotics may be the long-sought key in the treatment of PA keratitis/endophthalmitis. 

**Recommendation for experimental and clinical evaluation**

Although the hypothesis presented here is based on promising theoretical predictions and experimental studies, the authentic value of anti-pseudomonas nanoparticles in decreasing PA keratitis/endophthalmitis morbidities can only be evaluated in well-designed experimental animal models and subsequent clinical trials. Among anti-pseudomonas antibiotics, ceftazidime may be a good candidate in this regard, since reports of the successful preparation of its nano- and micrometric particles have been published recently [23]. Moreover, if one is not familiar with animal models of experimental PA keratitis/endophthalmitis, some good experimental models employed to quantitatively determine the bactericidal properties of new formulations or drugs can be readily found in the literature [24-27].

## CONCLUSION

If the present hypothesis holds, there is an effective complement, or even an alternative, to currently available topical antibiotics for the prevention of devastating sequels of PA keratitis. The main advantages of the suggested method would be less frequent topical applications of antibacterial therapies and, even more importantly, its ability to improve outcomes and potentially lessen the likelihood that infection progresses to more serious eye pathology. In the case of endophthalmitis, the implication would be that a non-invasive alternative, perhaps the topical application of nano-drugs, to the intraviterous administration of the antibiotics, is under way. This idea may also be noteworthy from a cost-effectiveness perspective as it may lead to the invention of a probably cheaper (by shortening the period of hospitalisation or by preventing the complications needing cornea transplantation) route of therapy for PA-related dilemma in ophthalmology.

## DISCLOSURE

The authors report no conflicts of interest in this work.
